# *Allium*-Based Phytobiotic Enhances Egg Production in Laying Hens through Microbial Composition Changes in Ileum and Cecum

**DOI:** 10.3390/ani11020448

**Published:** 2021-02-09

**Authors:** Miguel Rabelo-Ruiz, Juan José Ariza-Romero, María Jesús Zurita-González, Antonio Manuel Martín-Platero, Alberto Baños, Mercedes Maqueda, Eva Valdivia, Manuel Martínez-Bueno, Juan Manuel Peralta-Sánchez

**Affiliations:** 1 Departamento de Microbiología, Universidad de Granada, Avda. Fuentenueva, s/n, 18071 Granada, Spain; mrabelo@ugr.es (M.R.-R.); ammartin@ugr.es (A.M.M.-P.); mmaqueda@ugr.es (M.M.); evavm@ugr.es (E.V.); mmartine@ugr.es (M.M.-B.); 2 Departamento de Microbiología y Biotecnología, DMC Research Center, Camino de Jayena s/n, 18620 Granada, Spain; jariza@dmcrc.com (J.J.A.-R.); mayxu_93@hotmail.com (M.J.Z.-G.); abarjona@domca.com (A.B.); 3 Área de Endocrinología Molecular y Celular, Fundación Instituto de Investigación Sanotaria de Santiago de Compostela (FIDIS), Complejo Hospitalario Universitario De Santiago (CHUS), Servicio Gallego de Salud (SERGAS), 15706 Santiago de Compostela, Spain; 4 Instituto de Biotecnología, Universidad de Granada, Avda. Fuentenueva, s/n, 18071 Granada, Spain

**Keywords:** *Allium*-based phytobiotic, *Alliaceae* extract, laying hens, gut microbiota, egg production, high-throughput sequencing, Illumina MiSeq platform

## Abstract

**Simple Summary:**

The misuse of antibiotics has led several countries to ban their use as prophylactics against bacterial diseases or as growth promoters in livestock and poultry. Phytobiotics (bioactive compounds extracted from plants) are one of the alternatives, due to their antimicrobial activity and its modulation of the gut microbiota and the improvement of productive properties. Garlic and onion extracts, rich in antimicrobial compounds, are of the most promising alternative to antibiotics. We supplemented a garlic- and onion-based product in the diet to laying hens at the beginning of their productive life. The group supplied with this product produced in one month more eggs and with bigger size. This increase in production was accompanied by changes in the bacterial community of the gut. These changes in the microbiota suggest an improvement in food digestibility, as the most important changes produced by these compounds occur in the most distal parts of the gut. The relative abundance of beneficial *Lactococcus* in the ileum and *Lactobacillus* in the cecum increased in the experimental group. Both genera are known to have beneficial effects on host. These results are very promising for the use of these compounds in poultry for short periods.

**Abstract:**

Phytobiotics (bioactive compounds extracted from plants) are one of the explored alternatives to antibiotics in poultry and livestock due to their antimicrobial activity and its positive effects on gut microbiota and productive properties. In this study, we supplemented a product based on garlic and onion compounds in the diet to laying hens at the beginning of their productive life (from 16 to 20 weeks post-hatching). The experimental group showed a significant increase in the number of eggs laid and in their size, produced in one month compared to the control. This increase in production was accompanied by microbiota changes in the ileum and cecum by means of high throughput sequencing analyses. These bacterial shifts in the ileum were mainly the result of compositional changes in the rare biosphere (unweighted UniFrac), while in the cecum, treatment affected both majority and minority bacterial groups (weighted and unweighted UniFrac). These changes in the microbiota suggest an improvement in food digestibility. The relative abundance of *Lactococcus* in the ileum and *Lactobacillus* in the cecum increased significantly in the experimental group. The relative abundance of these bacterial genera are known to have positive effects on the hosts. These results are very promising for the use of these compounds in poultry for short periods.

## 1. Introduction

The abusive and inappropriate use of antibiotics in the animal production industry and clinical medicine has favored the selection of resistant bacteria and the spread of antibiotic resistance worldwide [1,2,3]. As a consequence, numerous countries have banned the use of most antibiotics as growth-promoters in livestock and poultry [4,5,6,7,8]. Some works predicted that these policies will increase production costs and final product prices [9,10], so the animal production industry is searching for efficient alternatives to the use of antibiotics as growth promoters in livestock, poultry and aquaculture [11,12,13]. Bacteriocins, bacteriophages, phytobiotics, probiotics, prebiotics and synbiotics have been proposed as the most promising alternatives [14,15].

Phytobiotics are bioactive compounds supplemented in the diet to improve the health and performance of farm animals [16,17]. Like antibiotics, phytobiotics can directly affect pathogenic bacteria, acting as antimicrobials [16,18] or by blocking some membrane receptors in pathogenic bacteria, making their adhesion to the intestinal mucosa difficult (reviewed in [16]). Phytobiotics can also act as prebiotics, supplying specific substrates and stimulating the growth of beneficial bacteria, or acting as growth-promoter metabolites [13,16]. Interestingly, phytobiotics may modulate the microbiota-gut-immune system, especially thorough antioxidant and anti-inflammation activities of these compounds [19]. Moreover, phytobiotics increase digestive enzyme activity, enhance feed conversion and hence improve the productive parameters of farm animals [17,19]. These improvements in digestive function have been related to the growth of beneficial bacteria in the cecum of broilers supplemented with phytobiotics, especially lactic acid bacteria such as lactobacilli and bifidobacteria [20,21]. These bacterial groups improve the host’s health by interacting with and training the immune system, allowing the host to allocate resources to production traits [22,23].

Extracts from plants of the *Alliaceae* family, mainly garlic (*Allium sativum*), onion (*Allium cepa*) and leek (*Allium porrum*) produce a wide variety of compounds showing antimicrobial activity, known of since ancient times [24,25,26]. The supplementation of these compounds has shown promising results in the health and productive parameters of several farm animals such as sheep [27], goats [28], cattle [29], pigs [30], broilers [31,32] and fishes [33]. These compounds improve intestinal functions such as rumen fermentations or energy-related blood metabolites, contributing to animal health and productivity [27,34]. In poultry, allium extracts supplemented in diets produce significant modulatory effects on growth, performance indices, lipid metabolism, gut ecosystem as well as immune responses, especially when poultry are experiencing stress and disease challenge conditions [35]. Most of these compounds are secondary metabolites, volatile organosulfur compounds, mainly thiosulfites and thiosulfonates which are responsible for the pungent odor, such as propyl thiosulfinate (PTS) and propyl propane thiosulfonate (PTSO) [24,36,37]. In in vitro experiments, these compounds showed anti-inflammatory properties in alveolar macrophages from pig lumps [36], antimicrobial activity against bacterial strains isolated from pig feces [37] and against Gram-negative and Gram-positive multidrug-resistant bacteria isolated from human fecal samples [38].

Previous works showed that experimental supplementation of garlic in the diet did not result in an increase in egg production [39,40,41,42], or even in decrease in egg productivity [43]. However, in a recent paper, Abad et al. [44] showed that a commercial *Allium* compound has positive effects on egg production. These differences in productivity effects due to Allium additives may be due to dose, duration of feeding or processing techniques [45]. In this manuscript, we hypothesize that the supplementation of *Allium* compounds in the diet of laying hens will affect bacterial community composition as well as egg production. We predict that a commercial *Alliaceae* extract supplemented in the diet of laying hens, similar to that used by Abad et al. [44] and based on PTS and PTSO, produces beneficial shifts in the gut microbiota and increase productive parameters, i.e., the number of laid eggs and their weights.

## 2. Materials and Methods

### 2.1. Experimental Animals and Facilities

The experiment was performed in 2014 at an experimental farm (Granja Avícola Gil, SL, Alhendín, Granada, Spain). Laying hens (Hy Line Brown) were placed in cages at the age of 16 weeks with food and water *ad libitum* and kept at 20 °C ± 2 °C and 78% ± 3% relative humidity (average ± standard deviation), under a photoperiod of 16 h per day throughout the experimental procedure.

### 2.2. Experimental Design and Sampling Collection

One hundred and eighty experimental laying hens were housed in groups of 6 hens per cage, with treatment groups being randomly distributed between production lines. Control hens (90 hens, 15 cages, 6 animals per cage) received a basal fodder diet (Supplementary Table S1), while experimental hens (90 hens, 15 cages, 6 animals per cage) received the same diet supplemented in feed with a commercial *Alliaceae* extract, Garlicon40 (DOMCA SAU, Granada, Spain) at a final concentration of 150 mg/kg (60 mg of PTSO per kg of feed).

The acclimation period lasted 15 days and then daily egg production (number of eggs) and their weight were recorded every working day (15 days of sampling). On day 30 after experiment started, 5 control and 8 experimental hens selected at random from different cages were euthanized by an intrathoracic injection of 2 mL/hen of T-61 (Intervet, Salamanca, Spain). Immediately after being slaughtered, the hens were dissected and the ileum and cecum were collected using sterile material. Each portion was homogenized in buffered peptone broth and aliquoted with 10% sucrose and finally frozen at −80 °C. Afterwards, the aliquots were lyophilized (LyoQuest, TELSTAR Technologies SL, Barcelona, Spain). No animal died during the experimental period due to illness or malnutrition.

### 2.3. High-throughput Sequencing

A total of 20 mg of lyophilized samples were used for bacterial DNA extraction from ileum and cecum samples using the QIAamp DNA Stool Mini Kit (QIAGEN). Amplicon libraries of the V4 region of the 16S rRNA gene were produced from total bacterial DNA by PCR amplification using primer pair 515f (5′-GTGCCAGCMGCCGCGGTAA-3′) and 786r (5´-GGACTACHVGGGTWTCTAAT-3′) with barcodes on the forward primer following Illumina library preparation (see Supplementary Materials and library preparation details in [46]). High-throughput sequencing was performed on Illumina MiSeq platform in the Scientific Instrumental Center at the University of Granada (Spain).

Subsequent analyses were performed with QIIME2 v2018.02 [47]. Primer trimming and pair joining were performed using default parameters. Afterwards, quality-filtering and sequence clustering were carried out using the Deblur algorithm, a sub-operational-taxonomic-unit approach that removes low quality sequences as well as sequencing errors [48], with a sequence length trimming limit of 252 base pairs. This algorithm allows an Amplicon Sequence Variant (ASV) table to be created. The fragment insertion script implemented in QIIME2 was used to perform a sequence alignment and construct a de-novo phylogenetic tree [49]. Taxonomy assignment was based on Greengenes 13_08 with a similarity of 99% [50]. Finally, non-bacterial sequences, i.e., chloroplasts and mitochondria, were removed from the sub-OTU table, although *Cyanobacteria* were retained in subsequent analyses [51].

### 2.4. Statistics

We used General Linear Models (GLM) to explore the effect of the treatment, sampling date and their interaction in number of eggs produced per day, mean egg size per day and alpha diversity indexes. For bacterial diversity analyses, we rarefied the ASV table at 2500 sequence depth per sample. We calculated different alpha diversity within sample diversity [52], indexes from the ASV table: bacterial OTU richness (or number of observed OTUs), evenness [53], Faith’s phylogenetic diversity index [54] and Shannon diversity index [55]. Residuals of the number of eggs, mean egg size and alpha diversity indexes after the analyses followed a normal distribution (Kolmogorov-Smirnov normality test; *p* > 0.20), validating the use of parametric statistical tests. These analyses were performed using STATISTICA 12.5 (Statsoft, Tulsa, OK, USA).

Difference in genera and phyla relative abundance between control and treated hens were explored by means of Linear Discriminant Analysis (LDA) Effect Size (LEfSe) [56]. LEfSe analyses were performed on the Galaxy web platform, run from a public server [57].

Weighted and unweighted UniFrac distance [58,59] were used to calculate beta diversity distance matrixes [differences between sample diversity, 52]. While weighted UniFrac gives more importance to the most abundant bacteria, unweighted UniFrac gives more importance to rare bacteria in the sub-OTUs as it takes into account their presence or absence regardless of abundance [59]. PERMANOVA was performed in order to test these effects on both UniFrac distance matrixes using PRIMER-7 software. Principal Coordinate Analyses were performed in order to visualize the first two PCoA axes using Emperor 2018.2.0 [60].

## 3. Results

### 3.1. Effect of Allium Supplementation on Egg Productivity of Laying Hens

**Figure 1:**
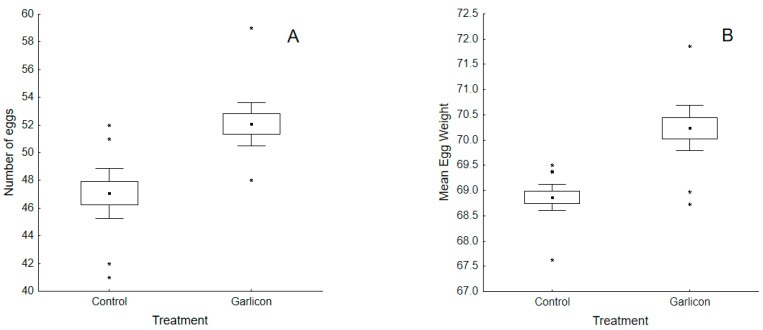
Differences in the mean number of eggs (**A**) and mean egg weight (**B**) produced by control and experimental laying hens. Control hens were fed a basal diet while experimental ones were fed a basal diet supplemented with a commercial *Alliaceae* extract. In both cases, the number of eggs and egg weight were significantly higher in the experimental group fed the *Alliaceae* extract. Whiskers show 95% confidence interval and asterisks indicate outliers. Outliers are represented with *.

**Figure 2:**
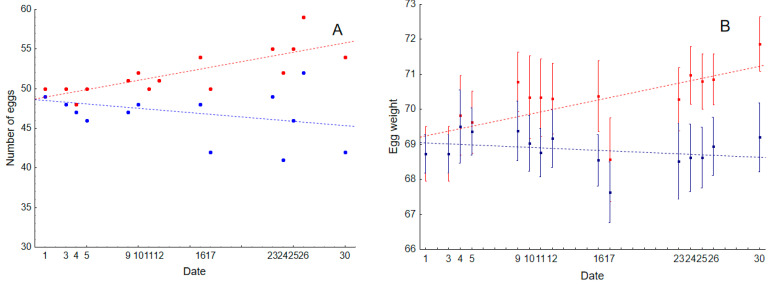
Changes in the number of eggs (**A**) and egg weight (**B**) produced by control laying hens (in blue) and laying hens experimentally supplemented with a commercial *Alliaceae* extract (in red) during 30 days of the experimental period. Regression lines and 95% CI are also shown.

Laying hens supplemented with the *Alliaceae* extract had higher egg production than the control group (Table 1, Figure 1A). While the control group decreased production throughout the experimental period in just 30 days (average egg number (standard error): 47.07 (0.79), the experimental group increased the number of eggs produced (52.07 (0.79); see interaction term in Table 1, Figure 2A).

The size of the eggs was also significantly affected by our experimental manipulation. Laying hens supplemented with the *Alliaceae* extract laid larger eggs (70.16 (0.18)) than the control group (68.85 (0.18)) (Table 1; Figure 1B). During the experimental period, egg weight in experimental hens increased, while egg size slightly decreased in the control group (see interaction term in Table 1, Figure 2B).

### 3.2. Changes in Bacterial Community Composition

**Figure 3:**
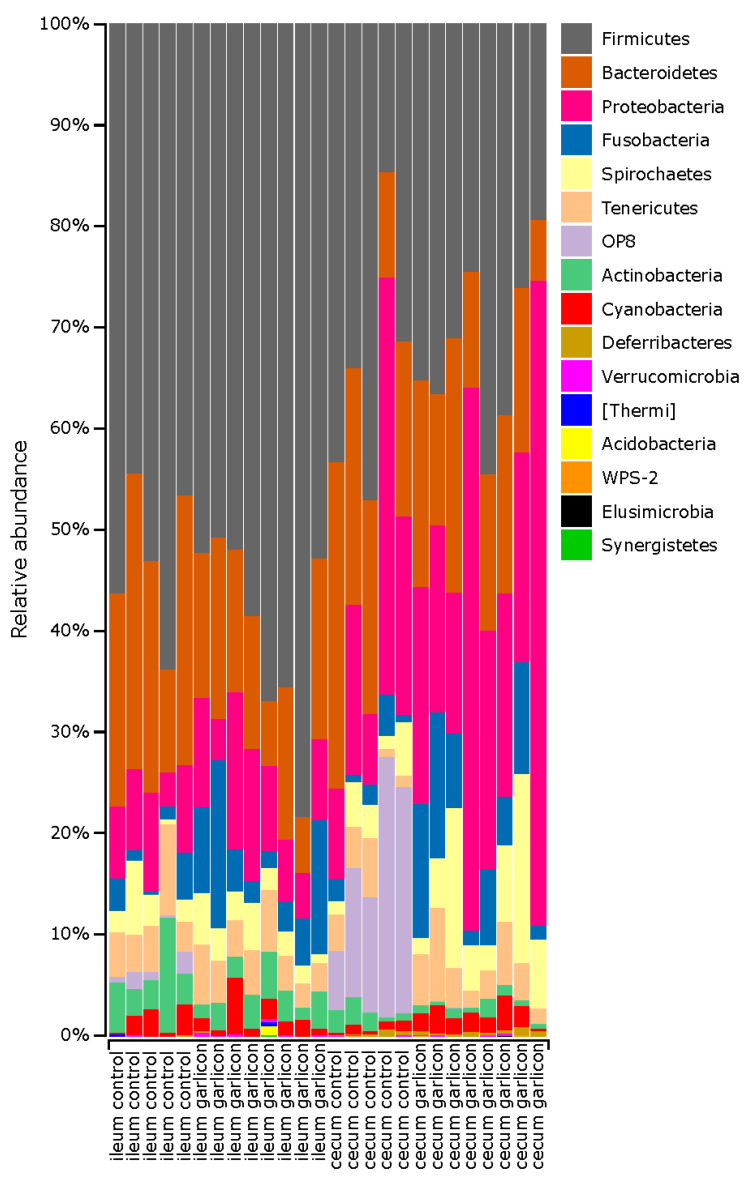
Bar plot of the relative bacterial abundance at the phylum level in different gut regions of laying hens and treatments. Control refers to laying hens fed a basal diet while Garlicon refers to experimental laying hens fed a basal diet supplemented with a commercial Alliaceae extract.

**Figure 4:**
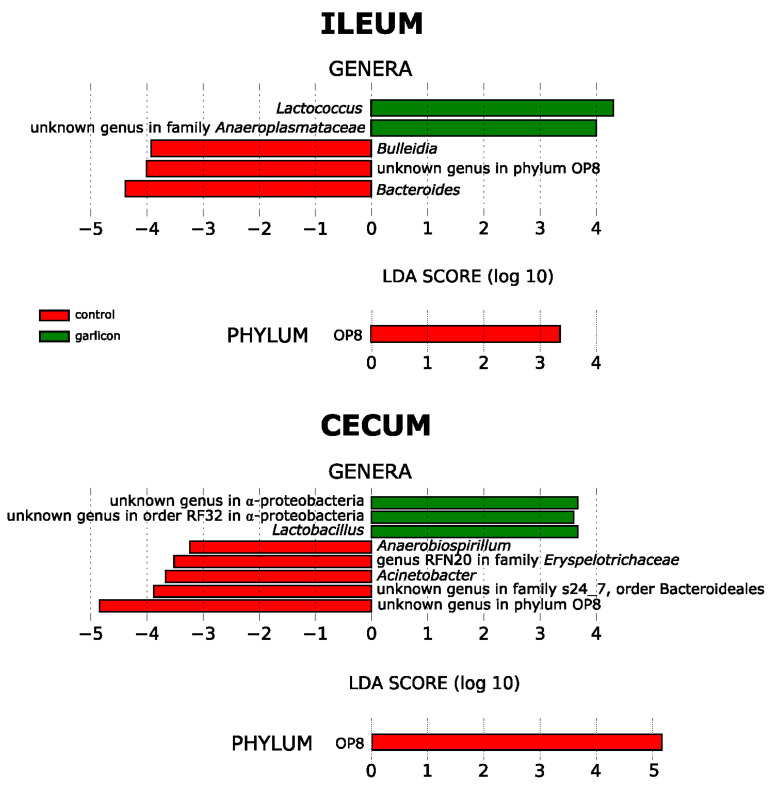
Linear Discriminant Analysis Effect Size (LEfSe) analyses showing bacterial genera (outer circles in the trees) and phyla (inner circles in the tree) that differed significantly between control hens and those supplemented with a commercial *Alliaceae* extract, in the ileum and cecum. Green bars and dots indicate a significant increase in relative abundance in the supplemented groups while red bars and dots showed a significant decrease.

The gut microbiota of laying hens is dominated by *Firmicutes, Bacteroidetes* and *Proteobacteria*. The relative abundance of these phyla depended on the gut region and treatment group (Figure 3). *Firmicutes* dominated in the ileum while *Proteobacteria* is dominant in the cecum. It is noteworthy that some minority phyla such as *Elusimicrobia* or *Synergistetes* decreased in the cecum of laying hens supplemented with the *Alliaceae* extract (Figure 3). However, only phylum OP8 differed significantly between treatments in both the ileum and cecum (Figure 4).

At the genus level, *Lactococcus* (*Firmicutes*) and an unidentified genus of *Anaeroplasmataceae* (*Tenericutes*) increased in the ileum while *Bulleidia* (*Firmicutes*), *Bacteroides* (*Bacteroidetes*) and an unknown genus of the phyla OP8 decreased in the supplemented hens (Figure 4, Supplementary Figures S1 and S2). In the cecum, two unknown genera of α-Proteobacteria and *Lactobacillus* (*Firmicutes*) increased significantly in the supplemented group. *Anaerobiospirillum* and *Acinetobacter* from *Gammaproteobacteria*, the genus RFN20 (*Firmicutes*), an unknown genus from *Bacteroidetes* and an unknown genus of OP8 decreased significantly in laying hens supplemented with the *Alliaceae* extract (Figure 4, Supplementary Figures S1 and S3).

### 3.3. Effect of Allium Compound Supplementation on Alpha and Beta Diversity

Supplementing the diet of laying hens with the *Alliaceae* extract did not affect any of the alpha diversity indexes after 30 days of treatment in any of the gut regions, ileum or cecum (Table 2).

**Figure 5:**
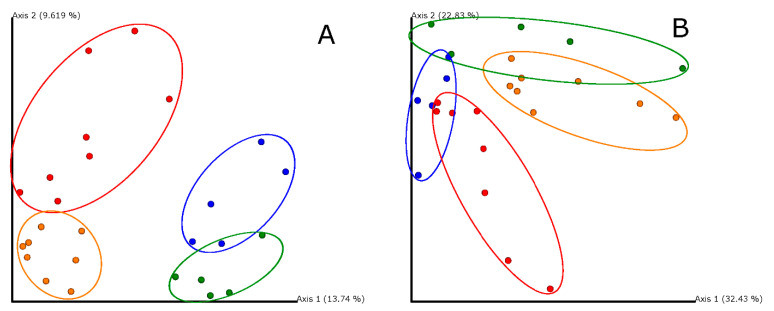
Principal Coordinate Analysis based in Unweighted UniFrac (**A**) and Weighted UniFrac (**B**) distance matrixes exploring the effects in the bacterial gut community of the supplementation with a commercial Alliaceae extract in laying hens’ diet (red: ileum, treated hens; orange: cecum, treated; blue: ileum, control; green: cecum, control). Circles surround samples from similar gut region and treatment. Percentages show the proportion of variance explained by each axis.

After 30 days of treatment, the bacterial community in laying hens varied significantly between the control and the supplemented hens (Table 3), in both the ileum and cecum samples, forming clear non-overlapping clusters (Figure 5). Interestingly, changes in the bacterial community between the ileum and cecum were similar between control and supplemented hens (see interaction terms in Table 3). Within each gut region, samples from the same treatment level clustered significantly together (Table 3, Figure 5), except for weighted UniFrac metrics in the ileum, showing a marginally significant trend (Table 3). Our experiment affected both, abundant and rare bacterial taxa, as it shows weighted and unweighted UniFrac results, respectively (Table 3, Figure 5).

## 4. Discussion

This study found that laying hens experimentally supplemented with *Allium* by-product compounds, based mainly on PTS and PTSO, significantly increased the number of laid eggs, as well as their size, after only 30 days of treatment. These productive increases in egg production and quality were accompanied by shifts in ileum and cecum microbiota, where some bacterial groups differed between the supplemented and the control group.

Phytobiotics have shown promising results in the health, performance and productivity of laying hens. Diet supplementation with *Allium* compounds possess beneficial effects: significantly reduces cholesterol levels in the plasma of laying hens [61,62,63,64], in egg contents [63] and even protects against several diseases, including cancer [65]. Supplementation with different phytobiotics such as black cumin seeds or leaves and extracts from the *Lamiaceae* family (such as peppermint, sage, rosemary, thyme and oregano) increase egg production and egg weight [64,66,67,68,69,70]. However, results for egg production in laying hens with their diet supplemented by *Allium* compounds are contradictory. Some studies pointed out the lack of effect on egg production or egg weight when hens were provided with different garlic preparations such as garlic paste, oil or powder [62,63,71]. However, and in accordance with our results, supplementation of garlic powder shows an increase in egg production [63]. Olobatoke and Mulugeta [72] found an increase in egg weight and a reduction in laying rate, but in laying hens supplemented with high doses of garlic powder. The differences in the associations between garlic-based compounds supplementation and these variables may be related with breeds of hen and the preparation and presentation of the garlic products [62,63,71], probably related to the composition and quantity of sulfur components [63].

Garlic, onion and its relatives are plants rich in several volatile organosulfur compounds responsible for the pungent odor and antimicrobial properties [24,71]. Allicin was one of the first compounds with antimicrobial activity to be isolated from garlic [73], although its instability does not allow it to be used in livestock and poultry [24]. Allicin has been substituted by other compounds in the use of *Allium*-derived substances in animal production and welfare, such as PTS and PTSO, by-products of the initial compounds present in garlic and onion such as alliin and propiin [37,74]. PTS is quite instable but it converts rapidly into PTSO, a more stable compound [37]. PTS and PTSO preparations have been shown to increase propionate concentrations in lamb rumen, improve weight gain and reduce non-esterified fatty acids and β-hydroxybutyrate [27]. Interestingly, high concentrations of garlic powder (5%) shows stronger garlic flavor in the eggs compared with those eggs laid by control and laying hens supplemented with lower doses (3%) [72]. In spite of this negative effects in organoleptic properties of eggs, PTSO did not seem to alter animal derived products. For instance, milk maintains its organoleptic properties after two months of PTSO supplementation in the diet of cows [29].

In broilers, supplementation of PTS and PTSO in the diet produces changes in the morphology and histology of the ileum and increases mucosa complexity in the gut [75]. It also produces shifts in the proximal intestinal microbiota of broilers, maintaining mucosal enzyme activity but improving food digestibility [76], with an associated increase in body weight [75]. These compounds also reduce *Salmonella* abundance in the ileum and *Escherichia coli* in the cecum of broilers [75]. Besides this direct effect of *Allium*-derived compounds, bacterial communities of the gut exclude pathogenic bacteria and enhance the development of the intestinal mucosa, epithelium and lamina propria, resulting in an improvement in farm animals’ health [77]. Reducing pathogenic bacteria brings relief to intestinal challenge and immune stress and hence the host can allocate resources to other traits [78,79]. In this sense, our results showed a significant shift in the bacterial community in the ileum and cecum in laying hens supplemented with *Allium*-derived compounds, as shown by the UniFrac analyses. Our results agree with previous findings where these changes in microbiota are especially important in the most distal parts of the gut of monogastric animals, such as the cecum [37]. The underlying action mechanisms of phytobiotics have not been explored yet, so it would deserve further studies, especially those related with changes in gut mucosa, the immune system and food digestibility.

Our results show that the relative abundance of *Lactococcus* in the ileum and *Lactobacillus* in the cecum increased significantly in laying hens supplemented with PTS and PTSO, while egg production improved. A recent paper based in culture-dependent techniques showed similar increase in egg production and fecal *Lactobacillus* counts in laying hens, supplemented with even lower doses of PTSO than the present work [44]. The increase in relative and absolute bacterial abundances of both *Lactococcus* and *Lactobacillus* produce beneficial effects in poultry and farm animals [80]. Han et al. [81] found that the relative abundance of *Lactococcus* in the cecum of broilers was positively correlated with body weight. Moreover, supplementation with a phytobiotic in laying hens increased *Lactobacillus* relative abundance in the cecum and simultaneously improved egg production as well as egg weights [68]. Most lactic acid bacteria have an intimate relationships with the health of their animal hosts [82], so these strains have been widely used as probiotics due to their many beneficial properties [83,84,85]. In this sense, these bacteria reduce the intestinal pH by producing lactic acid, and hence, inhibiting the proliferation of pathogenic bacteria (revised in [86]). The action of *Lactobacillus* may be also related with the reduction in the adhesion ability of *Salmonella* or of pathogenic bacteria as some strains of *Clostridium spp.* or *E. coli* [80]. Moreover, the levels of *Lactobacillus* could play a major role in promoting and maintaining intestinal inflammation, especially during inflammatory disease [80]. In this sense, lactic acid bacteria also increase the histological complexity of the gut and stimulate the immune response of the mucosa [86,87]. Despite not exploring the variables involved in these effects, a net positive effect in both lactic acid bacteria and egg production was found.

In animals, *Bacteroidetes* is present in the small and large intestine, although its relative abundance is much higher in the latter [82]. Our experimental procedure produced a significant reduction in the relative abundance of *Bacteroides* in the ileum and in an unidentified genus of the Order *Bacteroidales* in the cecum. *Bacteroidetes* species are involved in several metabolic activities in the gut, from carbohydrate fermentation to bile acid degradation (Bry et al., 1996; Phillips, 2009). Interestingly, Peinado et al. [88] found using qPCR that the absolute abundance of *Bacteroides* in Cobb broiler guts negatively correlated with *Lactobacillus* populations. In that study, they found an increase in *Bacteroides* and broiler performance in animals supplemented with PTSO while *Lactobacillus* abundance decreased. We can only speculate that these discrepancies may be due to the use of different molecular techniques (qPCR vs. high-throughput sequencing, absolute vs. relative abundances), different hen breeds (Cobb vs. Hy Line Brown) and more importantly, differences in age and sex. For instance, ileum microbiota differs significantly between male and female broilers only 3 days after hatching [89]. Moreover, age, sex and breed has a strong effect on bacterial community in the gut of broilers [90]. Further research is needed to clarify the effects of these confounding factors and explore new possibilities in broilers and laying hens as shown by the depletion of *Bacteroides* observed in obese children [91].

Our supplementation with PTS and PTSO depleted other genera that may cause negative effects on their hosts. *Acinetobacter* (*Moraxellaceae*, *Gammaproteobacteria*) is a common bacterium in soil environments and related with infections in immune-depressed patients [92]. *Anaerobiospirillum* (*Succinivibrionaceae*, *Gammaproteobacteria*) is a strict anaerobic genus causing septicemia and diarrhea in humans [93]. Some other taxa are poorly described so the effect of the reduction in abundance is unknown. These taxa include members of the *Erysipelotrichaceae* family, such as *Bulleida* and the genus *RFN20*, and an unidentified genus in the candidate phylum OP8. In this sense, *Aminicenantes*, the proposed name for this phylum, is poorly characterized and the few described strains cover a wide range of environments [94]. Similarly, other groups increased their abundance, such as a genus of *Anaeroplasmataceae*, anaerobic obligate commensals in the rumen of some mammals, the role of which has not yet been properly described [95] or the genus belonging to the Order RF32, the abundance of which correlates with histopathology and colonic inflammation in ray challenge with *E. coli*. Due to the lack of available information about the ecology and function of these groups in the gut, we cannot explain the significance of these changes in abundance in most of these strains in the treated hens. Culture-based methods are experiencing a rebirth in order to fill the gap in the knowledge of the huge amount of new microorganisms and diversity that next-generation technology is uncovering [96].

## 5. Conclusions

Our experimental supplementation of PTS and PTSO compounds in diet of laying hens increased their egg production and size, while producing shifts in the bacterial communities in the ileum and cecum of the hens. These results are very promising for the use of these phytobiotics in poultry for short periods (4 weeks in this study). Future research is necessary to understand the underlying mechanisms involved in these improvements, regarding the immune system, food digestibility and for longer exposition periods.

## Figures and Tables

**Table 1:** General Linear Models exploring the effects of treatment and date in egg productivity (Number of eggs) and size (mean egg size) of laying hens. Treated hens received a basal diet supplemented with a commercial *Alliaceae* extract. Significant values were set at 0.05, which are in bold. Degrees of freedom (d.f.) are also shown. * means interaction term.

	Model	Variables	d.f.	F	*p*
Egg number	1	Treatment	1,27	20.10	**<0.001**
		Date	1,27	1.07	0.310
		Treatment	1,26	<0.01	0.991
	2	Date	1,26	1.46	0.238
		Treatment * Date	1,26	10.82	**0.003**
Mean egg weight	3	Treatment	1,27	16.93	**<0.001**
		Date	1,27	2.89	0.089
		Treatment	1,26	0.04	0.840
	4	Date	1,26	2.44	0.119
		Treatment * Date	1,26	5.56	**0.019**

**Table 2:** General Linear Models exploring the effects of 30-days treatment in alpha diversity indexes in ileum and cecum of laying hens. Treated hens received in their basal diet supplemented with a commercial Alliaceae extract. Significant values were set at 0.05. Degrees of freedom (d.f.) are also shown.

	Alpha Diversity Index	Control	Experimental	d.f.	F	*p*
Ileum	sub-OUT richness	165.6 (13,12)	162.25 (10.37)	1,11	0.04	0.845
	Faith’s diversity index	20.92 (1.51)	20.56 (1.19)	1,11	0.03	0.857
	Evenness	0.79 (0.03)	0.74 (0.02)	1,11	2.20	0.167
	Shannon’s diversity index	5.79 (0.26)	5.40 (0.21)	1,11	1.41	0.260
Cecum	sub-OUT richness	173.20 (13.66)	172.12 (10.80)	1,11	<0.01	0.952
	Faith’s diversity index	21.34 (1.09)	20.96 (0.86)	1,11	0.08	0.789
	Evenness	0.67 (0.04)	0.69 (0.03)	1,11	0.18	0.682
	Shannon’s diversity index	4.97 (0.33)	5.11 (0.26)	1,11	0.12	0.737

**Table 3:** General Linear Models exploring the effects of 30-days treatment in beta diversity indexes in ileum and cecum of laying hens. Treated hens received a basal diet supplemented with a commercial *Alliaceae* extract. Significant values were set at 0.05, which are in bold. Degrees of freedom (d.f.) are also shown. * means interaction term.

	Beta Diversity Index	Factors	d.f.	F	*p*
Gut	Unweighted UniFfrac	Treatment	1,22	3.53	**<0.001**
		Gut	1,22	1.96	**0.002**
		Gut * Treatment	1,22	0.76	0.898
	Weighted UniFfrac	Treatment	1,22	4.27	**0.003**
		Gut	1,22	6.55	**<0.001**
		Gut * Treatment	1,22	1.03	0.391
Ileum	Unweighted UniFfrac	Treatment	1,11	1.81	**<0.001**
	Weighted UniFfrac	Treatment	1,11	2.21	0.056
Cecum	Unweighted UniFfrac	Treatment	1,11	2.63	**0.002**
	Weighted Unifrac	Treatment	1,11	3.11	**0.020**

## Data Availability

Sequences are available in the Sequence Read Archive (SRA) in the GenBank-NCBI webpage1 under Accession Nos. SAMN09603288 to SAMN9603307 and SAMN09603326 to SAMN 09603341.

## Supplementary Materials

The following are available online at https://www.mdpi.com/2076-2615/11/2/448/s1; Figure S1. Bar plot of the relative bacterial abundance at the genus level in different gut regions of laying hens and treatments. Control refers to laying hens fed a basal diet while Garlicon refers to experimental laying hens fed a basal diet supplemented with the commercial *Alliaceae* extract. The sixteen most abundance genera are shown in a unique color set. The color of the rest of genera (less abundant) are repeated every 8 colors; Figure S2. Linear Discriminant Analysis Effect Size (Lefse) showing genera from the ileum that significantly differ between control and experimentally supplemented with a commercial Alliaceae laying hens. Bars showed relative abundance of the genus in each sample. Solid line represents mean relative abundance while dashed line represent the median. k: kingdom; p: phylum; c: class; o: order; f: family; and g: genus; Figure S3. Linear Discriminant Analysis Effect Size (Lefse) showing genera from the cecum (*ciego*) that significantly differ between control and experimentally supplemented with a commercial Alliaceae laying hens. Bars showed relative abundance of the genus in each sample. Solid line denotes relative abundance while dashed line showed median relative abundance. k: kingdom; p: phylum; c: class; o: order; f: family; and g: genus; Table S1: Nutritional information of the basal feed employed in laying hens.
