# Supplementary material for: Allium-Based Phytobiotic Enhances Egg Production in Laying Hens through Microbial Composition Changes in Ileum and Cecum
Source: Animals (Basel). 2021 Feb 9;11(2):448. doi: 10.3390/ani11020448 (PMC7915398; doi:10.3390/ani11020448)
Supplement: Supplementary file 1 [file animals-11-00448-s001.pdf]

Table S1. Nutritional information of the basal feed employed in laying hens.

	%
Raw protein	16.00
Carbohydrates	57.57
Raw fiber	3.90
Fat content	3.50
Ashes	13.00
Calcium	3.85
Phosphorus	0.59
Sodium	0.16
Lysine	0.97
Metionine	0.46

Supplementary Figures 1. Bar plot of the relative bacterial abundance at the genus level in different gut regions of laying hens and treatments. Control refers to laying hens fed a basal diet while Garlicon refers to experimental laying hens fed a basal diet supplemented with the commercial *Alliaceae* extract. The sixteen most abundance genera are shown in a unique color set. The color of the rest of genera (less abundant) are repeated every 8 colors.

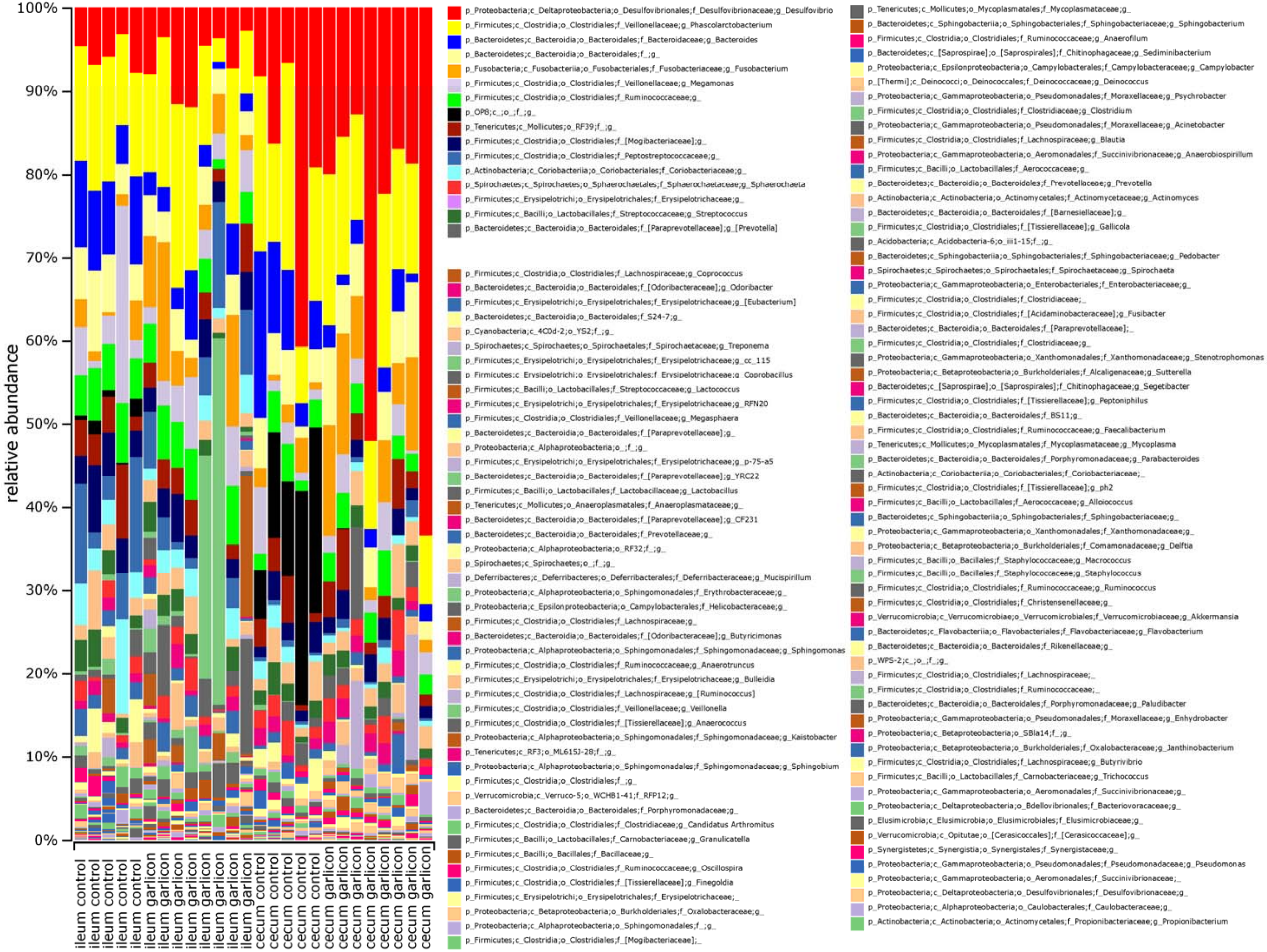
100%
90%
80%
70%
60%
50%
40%
30%
20%
10%
0%
relative abundance
ileum control
ileum control
ileum control
ileum control
ileum control
ileum garlicon
ileum garlicon
ileum garlicon
ileum garlicon
ileum garlicon
ileum garlicon
ileum garlicon
ileum garlicon
cecum control
cecum control
cecum control
cecum control
cecum control
cecum garlicon
cecum garlicon
cecum garlicon
cecum garlicon
cecum garlicon
cecum garlicon
cecum garlicon
cecum garlicon
p_Proteobacteria;c_Deltaproteobacteria;o_Desulfovibrionales;f_Desulfovibrionaceae;g_Desulfovibrio
p_Firmicutes;c_Clostridia;o_Clostridiales;f_Veillonellaceae;g_Phascolarctobacterium
p_Bacteroidetes;c_Bacteroidia;o_Bacteroidales;f_Bacteroidaceae;g_Bacteroides
p_Bacteroidetes;c_Bacteroidia;o_Bacteroidales;f_;g_
p_Fusobacteria;c_Fusobacteriia;o_Fusobacteriales;f_Fusobacteriaceae;g_Fusobacterium
p_Firmicutes;c_Clostridia;o_Clostridiales;f_Veillonellaceae;g_Megamonas
p_Firmicutes;c_Clostridia;o_Clostridiales;f_Ruminococcaceae;g_
p_OP8;c_;o_;f_;g_
p_Tenericutes;c_Mollicutes;o_RF39;f_;g_
p_Firmicutes;c_Clostridia;o_Clostridiales;f_[Mogibacteriaceae];g_
p_Firmicutes;c_Clostridia;o_Clostridiales;f_Peptostreptococcaceae;g_
p_Actinobacteria;c_Coriobacteriia;o_Coriobacteriales;f_Coriobacteriaceae;g_
p_Spirochaetes;c_Spirochaetes;o_Sphaerochaetales;f_Sphaerochaetaceae;g_Sphaerochaeta
p_Firmicutes;c_Erysipelotrichi;o_Erysipelotrichales;f_Erysipelotrichaceae;g_
p_Firmicutes;c_Bacilli;o_Lactobacillales;f_Streptococcaceae;g_Streptococcus
p_Bacteroidetes;c_Bacteroidia;o_Bacteroidales;f_[Paraprevotellaceae];g_[Prevotella]
p_Firmicutes;c_Clostridia;o_Clostridiales;f_Lachnospiraceae;g_Coprococcus
p_Bacteroidetes;c_Bacteroidia;o_Bacteroidales;f_[Odoribacteraceae];g_Odoribacter
p_Firmicutes;c_Erysipelotrichi;o_Erysipelotrichales;f_Erysipelotrichaceae;g_[Eubacterium]
p_Bacteroidetes;c_Bacteroidia;o_Bacteroidales;f_S24-7;g_
p_Cyanobacteria;c_4C0d-2;o_YS2;f_;g_
p_Spirochaetes;c_Spirochaetes;o_Spirochaetales;f_Spirochaetaceae;g_Treponema
p_Firmicutes;c_Erysipelotrichi;o_Erysipelotrichales;f_Erysipelotrichaceae;g_cc_115
p_Firmicutes;c_Erysipelotrichi;o_Erysipelotrichales;f_Erysipelotrichaceae;g_Coprobacillus
p_Firmicutes;c_Bacilli;o_Lactobacillales;f_Streptococcaceae;g_Lactococcus
p_Firmicutes;c_Erysipelotrichi;o_Erysipelotrichales;f_Erysipelotrichaceae;g_RFN20
p_Firmicutes;c_Clostridia;o_Clostridiales;f_Veillonellaceae;g_Megasphaera
p_Bacteroidetes;c_Bacteroidia;o_Bacteroidales;f_[Paraprevotellaceae];g_
p_Proteobacteria;c_Alphaproteobacteria;o_;f_;g_
p_Firmicutes;c_Erysipelotrichi;o_Erysipelotrichales;f_Erysipelotrichaceae;g_p-75-a5
p_Bacteroidetes;c_Bacteroidia;o_Bacteroidales;f_[Paraprevotellaceae];g_YRC22
p_Firmicutes;c_Bacilli;o_Lactobacillales;f_Lactobacillaceae;g_Lactobacillus
p_Tenericutes;c_Mollicutes;o_Anaeroplasmatales;f_Anaeroplasmataceae;g_
p_Bacteroidetes;c_Bacteroidia;o_Bacteroidales;f_[Paraprevotellaceae];g_CF231
p_Bacteroidetes;c_Bacteroidia;o_Bacteroidales;f_Prevotellaceae;g_
p_Proteobacteria;c_Alphaproteobacteria;o_RF32;f_;g_
p_Spirochaetes;c_Spirochaetes;o_;f_;g_
p_Deferribacteres;c_Deferribacteres;o_Deferribacterales;f_Deferribacteraceae;g_Mucispirillum
p_Proteobacteria;c_Alphaproteobacteria;o_Sphingomonadales;f_Erythrobacteraceae;g_
p_Proteobacteria;c_Epsilonproteobacteria;o_Campylobacterales;f_Helicobacteraceae;g_
p_Firmicutes;c_Clostridia;o_Clostridiales;f_Lachnospiraceae;g_
p_Bacteroidetes;c_Bacteroidia;o_Bacteroidales;f_[Odoribacteraceae];g_Butyricimonas
p_Proteobacteria;c_Alphaproteobacteria;o_Sphingomonadales;f_Sphingomonadaceae;g_Sphingomonas
p_Firmicutes;c_Clostridia;o_Clostridiales;f_Ruminococcaceae;g_Anaerotruncus
p_Firmicutes;c_Erysipelotrichi;o_Erysipelotrichales;f_Erysipelotrichaceae;g_Bulleidia
p_Firmicutes;c_Clostridia;o_Clostridiales;f_Lachnospiraceae;g_[Ruminococcus]
p_Firmicutes;c_Clostridia;o_Clostridiales;f_Veillonellaceae;g_Veillonella
p_Firmicutes;c_Clostridia;o_Clostridiales;f_[Tissierellaceae];g_Anaerococcus
p_Proteobacteria;c_Alphaproteobacteria;o_Sphingomonadales;f_Sphingomonadaceae;g_Kaistobacter
p_Tenericutes;c_RF3;o_ML615J-28;f_;g_
p_Proteobacteria;c_Alphaproteobacteria;o_Sphingomonadales;f_Sphingomonadaceae;g_Sphingobium
p_Firmicutes;c_Clostridia;o_Clostridiales;f_;g_
p_Verrucomicrobia;c_Verruco-5;o_WCHB1-41;f_RFP12;g_
p_Bacteroidetes;c_Bacteroidia;o_Bacteroidales;f_Porphyromonadaceae;g_
p_Firmicutes;c_Clostridia;o_Clostridiales;f_Clostridiaceae;g_Candidatus Arthromitus
p_Firmicutes;c_Bacilli;o_Lactobacillales;f_Carnobacteriaceae;g_Granulicatella
p_Firmicutes;c_Bacilli;o_Bacillales;f_Bacillaceae;g_
p_Firmicutes;c_Clostridia;o_Clostridiales;f_Ruminococcaceae;g_Oscillospira
p_Firmicutes;c_Clostridia;o_Clostridiales;f_[Tissierellaceae];g_Finegoldia
p_Firmicutes;c_Erysipelotrichi;o_Erysipelotrichales;f_Erysipelotrichaceae;_
p_Proteobacteria;c_Betaproteobacteria;o_Burkholderiales;f_Oxalobacteraceae;g_
p_Proteobacteria;c_Alphaproteobacteria;o_Sphingomonadales;f_;g_
p_Firmicutes;c_Clostridia;o_Clostridiales;f_[Mogibacteriaceae];_
p_Tenericutes;c_Mollicutes;o_Mycoplasmatales;f_Mycoplasmataceae;g_
p_Bacteroidetes;c_Sphingobacteriia;o_Sphingobacteriales;f_Sphingobacteriaceae;g_Sphingobacterium
p_Firmicutes;c_Clostridia;o_Clostridiales;f_Ruminococcaceae;g_Anaerofilum
p_Bacteroidetes;c_[Saprospirae];o_[Saprospirales];f_Chitinophagaceae;g_Sediminibacterium
p_Proteobacteria;c_Epsilonproteobacteria;o_Campylobacterales;f_Campylobacteraceae;g_Campylobacter
p_[Thermi];c_Deinococci;o_Deinococcales;f_Deinococcaceae;g_Deinococcus
p_Proteobacteria;c_Gammaproteobacteria;o_Pseudomonadales;f_Moraxellaceae;g_Psychrobacter
p_Firmicutes;c_Clostridia;o_Clostridiales;f_Clostridiaceae;g_Clostridium
p_Proteobacteria;c_Gammaproteobacteria;o_Pseudomonadales;f_Moraxellaceae;g_Acinetobacter
p_Firmicutes;c_Clostridia;o_Clostridiales;f_Lachnospiraceae;g_Blautia
p_Proteobacteria;c_Gammaproteobacteria;o_Aeromonadales;f_Succinivibrionaceae;g_Anaerobiospirillum
p_Firmicutes;c_Bacilli;o_Lactobacillales;f_Aerococcaceae;g_
p_Bacteroidetes;c_Bacteroidia;o_Bacteroidales;f_Prevotellaceae;g_Prevotella
p_Actinobacteria;c_Actinobacteria;o_Actinomycetales;f_Actinomycetaceae;g_Actinomyces
p_Bacteroidetes;c_Bacteroidia;o_Bacteroidales;f_[Barnesiellaceae];g_
p_Firmicutes;c_Clostridia;o_Clostridiales;f_[Tissierellaceae];g_Gallicola
p_Acidobacteria;c_Acidobacteria-6;o_iii1-15;f_;g_
p_Bacteroidetes;c_Sphingobacteriia;o_Sphingobacteriales;f_Sphingobacteriaceae;g_Pedobacter
p_Spirochaetes;c_Spirochaetes;o_Spirochaetales;f_Spirochaetaceae;g_Spirochaeta
p_Proteobacteria;c_Gammaproteobacteria;o_Enterobacteriales;f_Enterobacteriaceae;g_
p_Firmicutes;c_Clostridia;o_Clostridiales;f_Clostridiaceae;_
p_Firmicutes;c_Clostridia;o_Clostridiales;f_[Acidaminobacteraceae];g_Fusibacter
p_Bacteroidetes;c_Bacteroidia;o_Bacteroidales;f_[Paraprevotellaceae];_
p_Firmicutes;c_Clostridia;o_Clostridiales;f_Clostridiaceae;g_
p_Proteobacteria;c_Gammaproteobacteria;o_Xanthomonadales;f_Xanthomonadaceae;g_Stenotrophomonas
p_Proteobacteria;c_Betaproteobacteria;o_Burkholderiales;f_Alcaligenaceae;g_Sutterella
p_Bacteroidetes;c_[Saprospirae];o_[Saprospirales];f_Chitinophagaceae;g_Segetibacter
p_Firmicutes;c_Clostridia;o_Clostridiales;f_[Tissierellaceae];g_Peptoniphilus
p_Bacteroidetes;c_Bacteroidia;o_Bacteroidales;f_BS11;g_
p_Firmicutes;c_Clostridia;o_Clostridiales;f_Ruminococcaceae;g_Faecalibacterium
p_Tenericutes;c_Mollicutes;o_Mycoplasmatales;f_Mycoplasmataceae;g_Mycoplasma
p_Bacteroidetes;c_Bacteroidia;o_Bacteroidales;f_Porphyromonadaceae;g_Parabacteroides
p_Actinobacteria;c_Coriobacteriia;o_Coriobacteriales;f_Coriobacteriaceae;_
p_Firmicutes;c_Clostridia;o_Clostridiales;f_[Tissierellaceae];g_ph2
p_Firmicutes;c_Bacilli;o_Lactobacillales;f_Aerococcaceae;g_Alloiococcus
p_Bacteroidetes;c_Sphingobacteriia;o_Sphingobacteriales;f_Sphingobacteriaceae;g_
p_Proteobacteria;c_Gammaproteobacteria;o_Xanthomonadales;f_Xanthomonadaceae;g_
p_Proteobacteria;c_Betaproteobacteria;o_Burkholderiales;f_Comamonadaceae;g_Delftia
p_Firmicutes;c_Bacilli;o_Bacillales;f_Staphylococcaceae;g_Macrococcus
p_Firmicutes;c_Bacilli;o_Bacillales;f_Staphylococcaceae;g_Staphylococcus
p_Firmicutes;c_Clostridia;o_Clostridiales;f_Ruminococcaceae;g_Ruminococcus
p_Firmicutes;c_Clostridia;o_Clostridiales;f_Christensenellaceae;g_
p_Verrucomicrobia;c_Verrucomicrobiae;o_Verrucomicrobiales;f_Verrucomicrobiaceae;g_Akkermansia
p_Bacteroidetes;c_Flavobacteriia;o_Flavobacteriales;f_Flavobacteriaceae;g_Flavobacterium
p_Bacteroidetes;c_Bacteroidia;o_Bacteroidales;f_Rikenellaceae;g_
p_WPS-2;c_;o_;f_;g_
p_Firmicutes;c_Clostridia;o_Clostridiales;f_Lachnospiraceae;_
p_Firmicutes;c_Clostridia;o_Clostridiales;f_Ruminococcaceae;_
p_Bacteroidetes;c_Bacteroidia;o_Bacteroidales;f_Porphyromonadaceae;g_Paludibacter
p_Proteobacteria;c_Gammaproteobacteria;o_Pseudomonadales;f_Moraxellaceae;g_Enhydrobacter
p_Proteobacteria;c_Betaproteobacteria;o_SBla14;f_;g_
p_Proteobacteria;c_Betaproteobacteria;o_Burkholderiales;f_Oxalobacteraceae;g_Janthinobacterium
p_Firmicutes;c_Clostridia;o_Clostridiales;f_Lachnospiraceae;g_Butyrivibrio
p_Firmicutes;c_Bacilli;o_Lactobacillales;f_Carnobacteriaceae;g_Trichococcus
p_Proteobacteria;c_Gammaproteobacteria;o_Aeromonadales;f_Succinivibrionaceae;g_
p_Proteobacteria;c_Deltaproteobacteria;o_Bdellovibrionales;f_Bacteriovoracaceae;g_
p_Elusimicrobia;c_Elusimicrobia;o_Elusimicrobiales;f_Elusimicrobiaceae;g_
p_Verrucomicrobia;c_Opitutae;o_[Cerasicoccales];f_[Cerasicoccaceae];g_
p_Synergistetes;c_Synergistia;o_Synergistales;f_Synergistaceae;g_
p_Proteobacteria;c_Gammaproteobacteria;o_Pseudomonadales;f_Pseudomonadaceae;g_Pseudomonas
p_Proteobacteria;c_Gammaproteobacteria;o_Aeromonadales;f_Succinivibrionaceae;_
p_Proteobacteria;c_Deltaproteobacteria;o_Desulfovibrionales;f_Desulfovibrionaceae;g_
p_Proteobacteria;c_Alphaproteobacteria;o_Caulobacterales;f_Caulobacteraceae;g_
p_Actinobacteria;c_Actinobacteria;o_Actinomycetales;f_Propionibacteriaceae;g_Propionibacterium

Figure S2. Linear Discriminant Analysis Effect Size (Lefse) showing genera from the ileum that significantly differ between control and experimentally supplemented with a commercial Alliaceae (Garlicon40 ©) laying hens. Bars showed relative abundance of the genus in each sample. Solid line represents mean relative abundance while dashed line represent the median. k: kingdom; p: phylum; c: class; o: order; f: family; and g: genus.

k__Bacteria_p__Bacteroidetes_c__Bacteroidia_o__Bacteroidales_f__Bacteroidaceae_g__Bacteroides

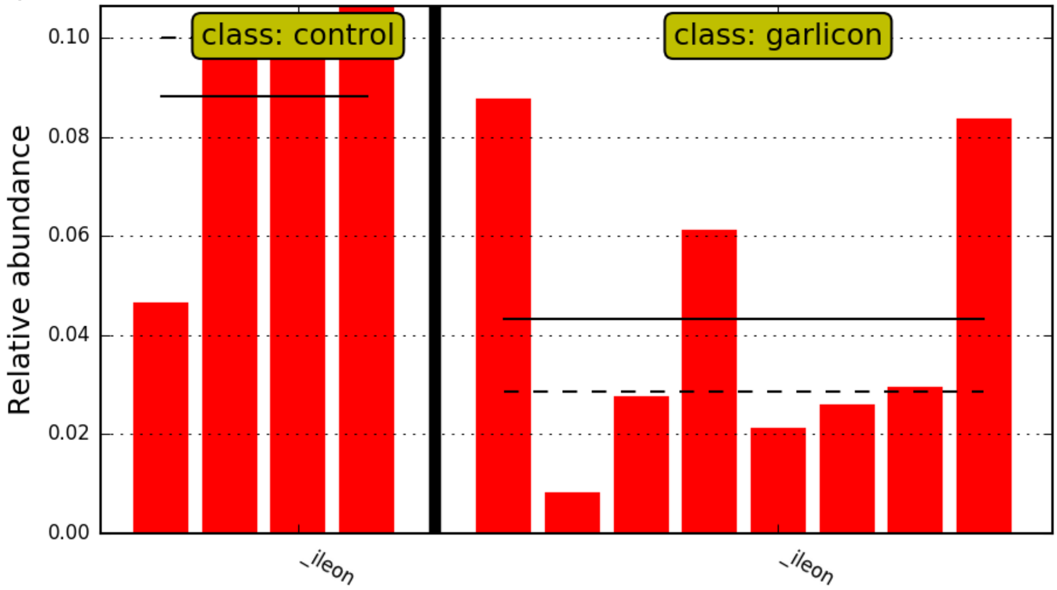

k__Bacteria_p__Firmicutes_c__Bacilli_o__Lactobacillales_f__Streptococcaceae_g__Lactococcus

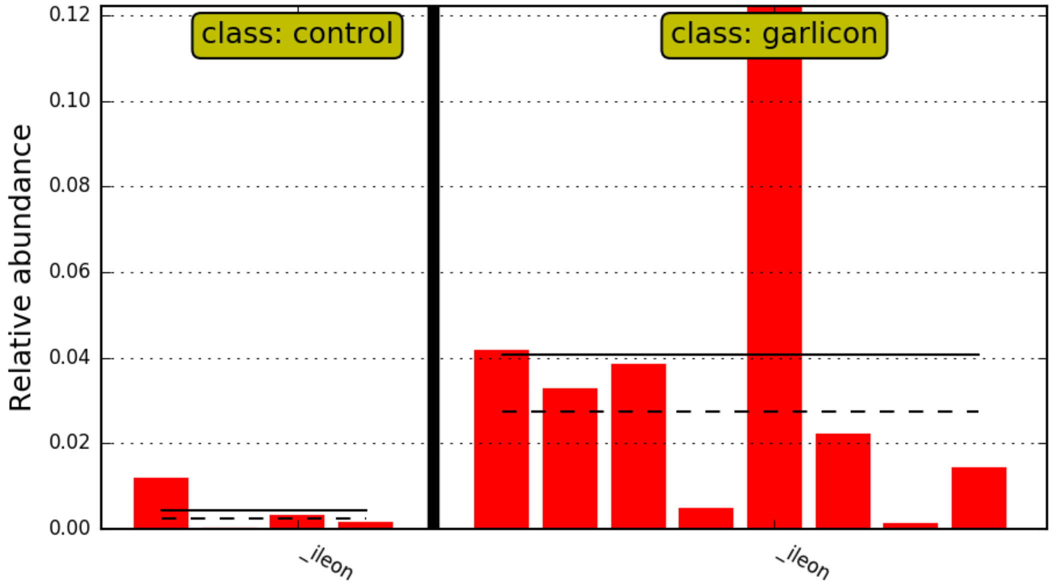

k__Bacteria_p__Firmicutes_c__Erysipelotrichi_o__Erysipelotrichales_f__Erysipelotrichaceae_g__Bulleidia

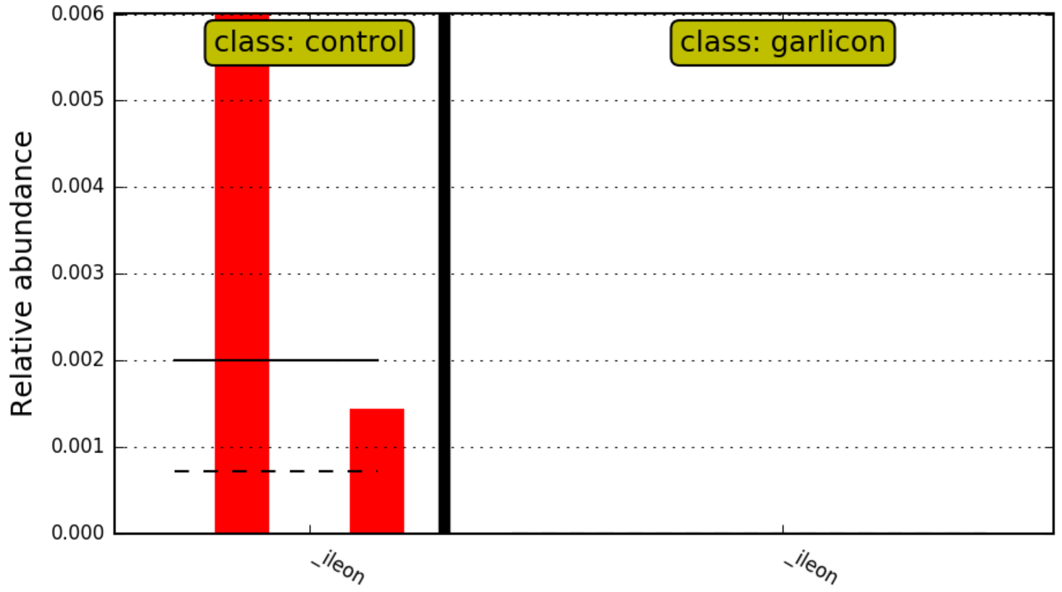

k__Bacteria_p__OP8_c___o___f___g__

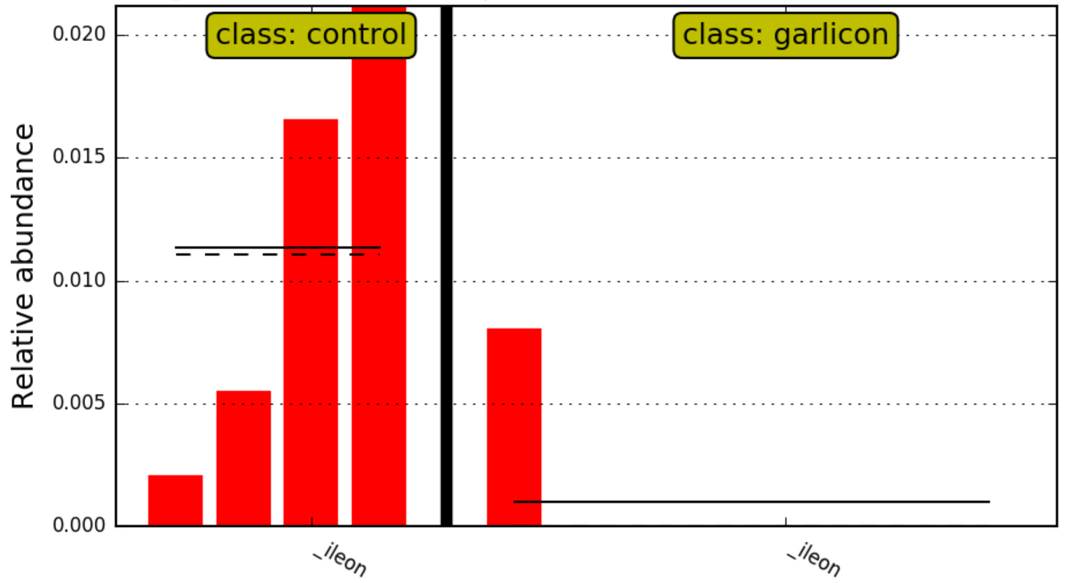

k__Bacteria_p__Tenericutes_c__Mollicutes_o__Anaeroplasmatales_f__Anaeroplasmataceae_g__

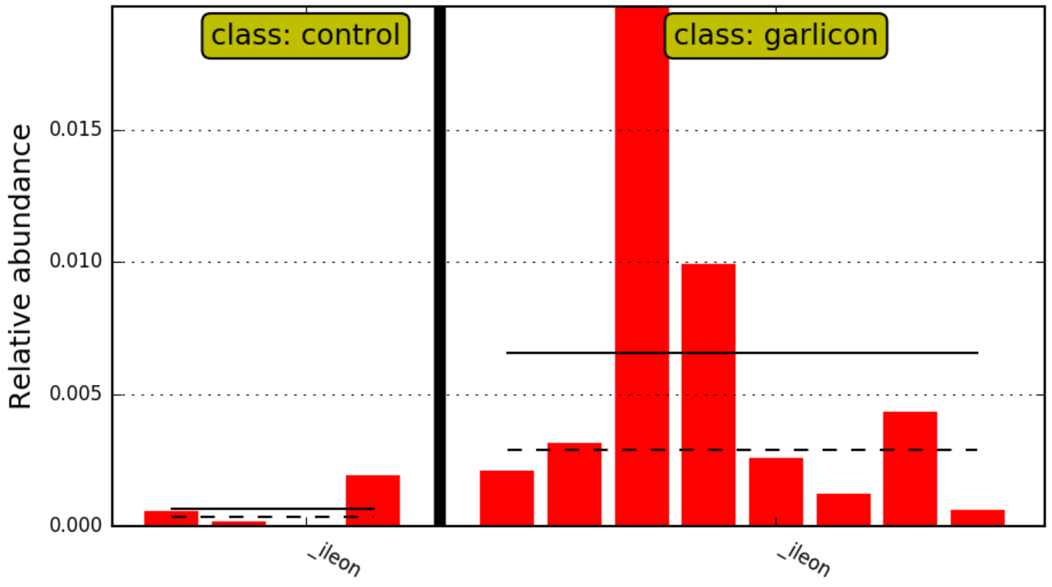
class: control
class: garlicon
Relative abundance
0.015
0.010
0.005
0.000
_ileon
_ileon

Figure S3. Linear Discriminant Analysis Effect Size (Lefse) showing genera from the cecum (*ciego*) that significantly differ between control and experimentally supplemented with a commercial Alliaceae (Garlicon40 ©) laying hens. Bars showed relative abundance of the genus in each sample. Solid line mean relative abundance while dashed line showed median relative abundance. k: kingdom; p: phylum; c: class; o: order; f: family; and g: genus.

k__Bacteria_p__Bacteroidetes_c__Bacteroidia_o__Bacteroidales_f__S24_7_g__

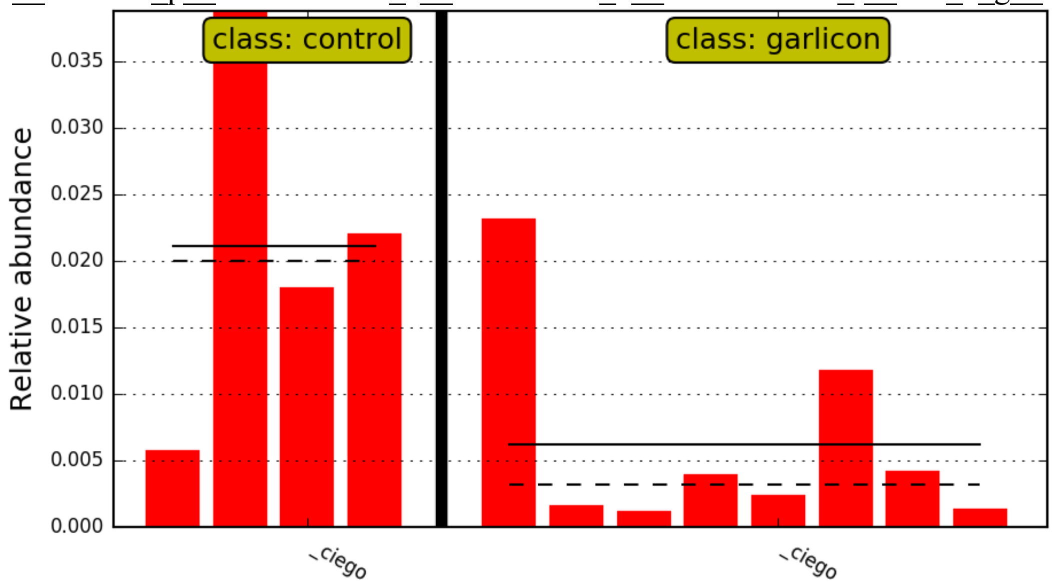

k__Bacteria_p__Firmicutes_c__Bacilli_o__Lactobacillales_f__Lactobacillaceae_g__Lactobacillus

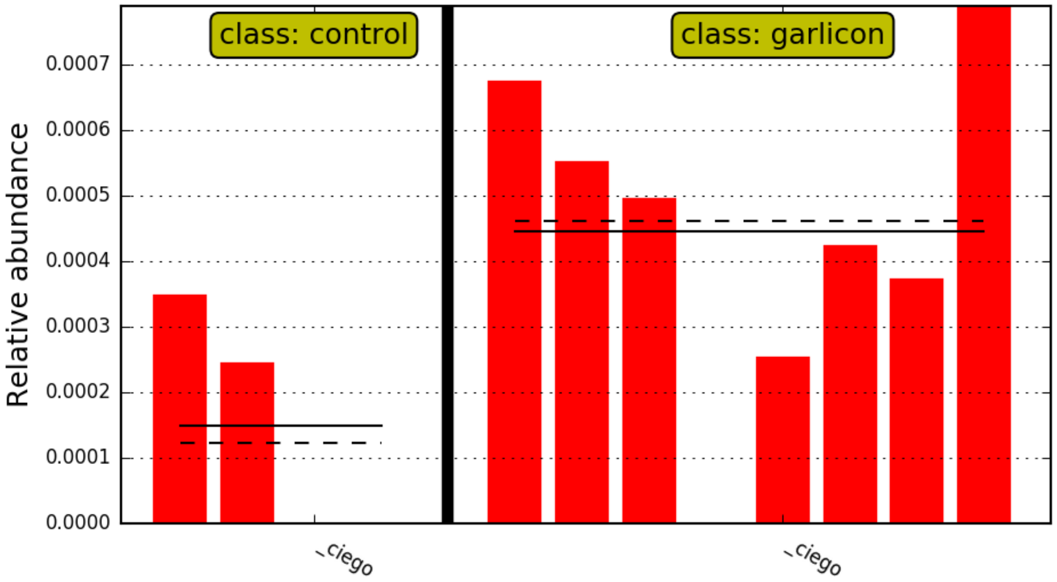

k__Bacteria_p__Firmicutes_c__Erysipelotrichi_o__Erysipelotrichales_f__Erysipelotrichaceae_g__RFN20

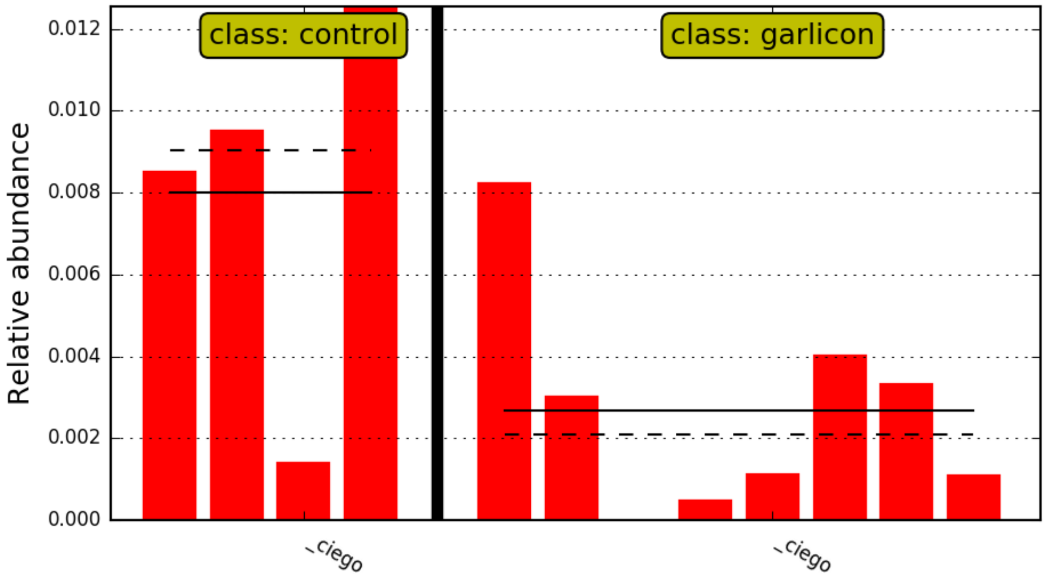

k__Bacteria_p__OP8_c___o___f___g__

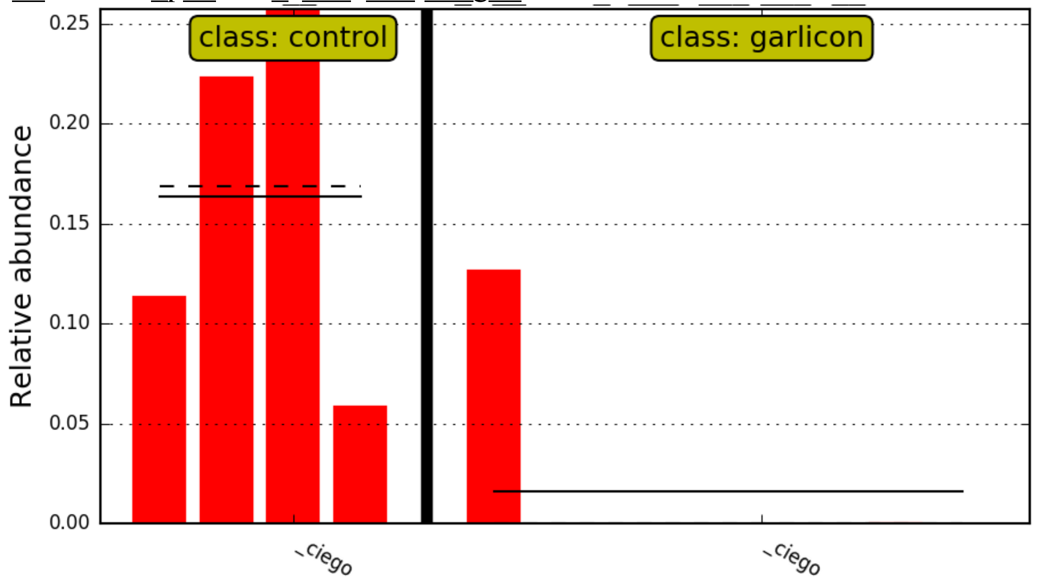

k__Bacteria_p__Proteobacteria_c__Alphaproteobacteria_o___f___g__

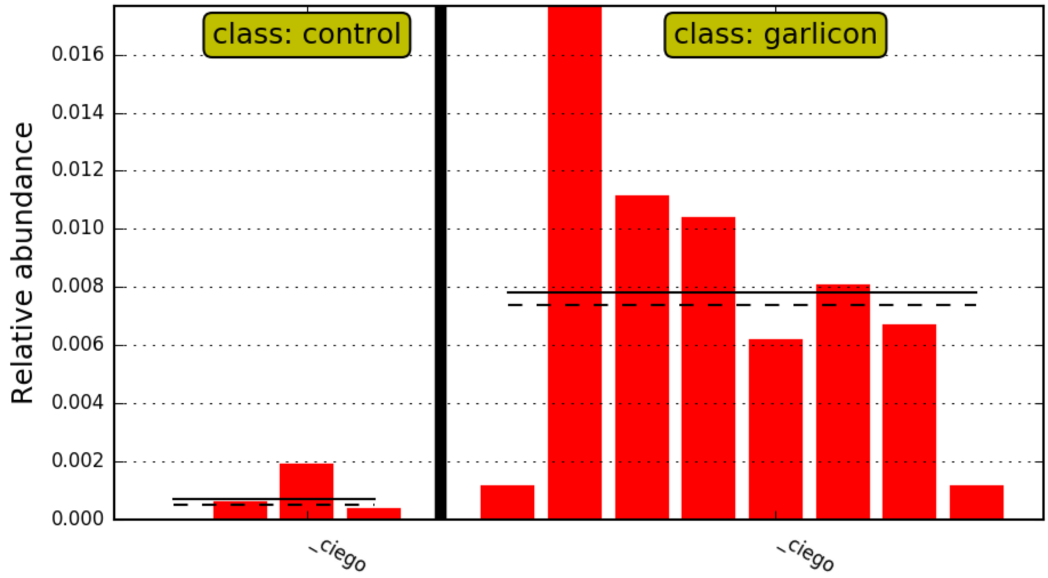

k__Bacteria_p__Proteobacteria_c__Alphaproteobacteria_o__RF32_f__g__

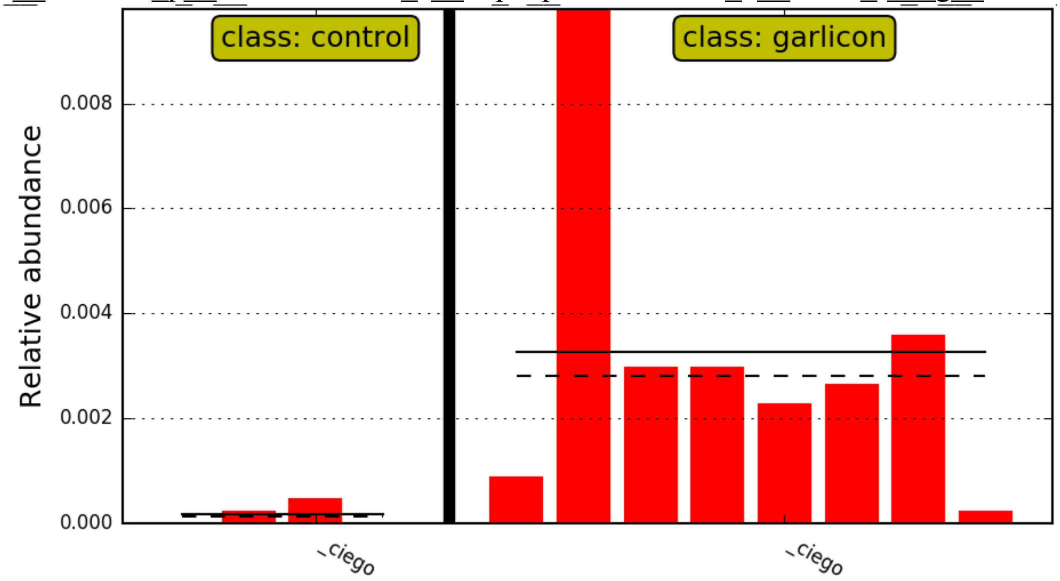

k__Bacteria_p__Proteobacteria_c__Gammaproteobacteria_o__Aeromonadales_f__Succinivibrionaceae_g__Anaerobiospirillum

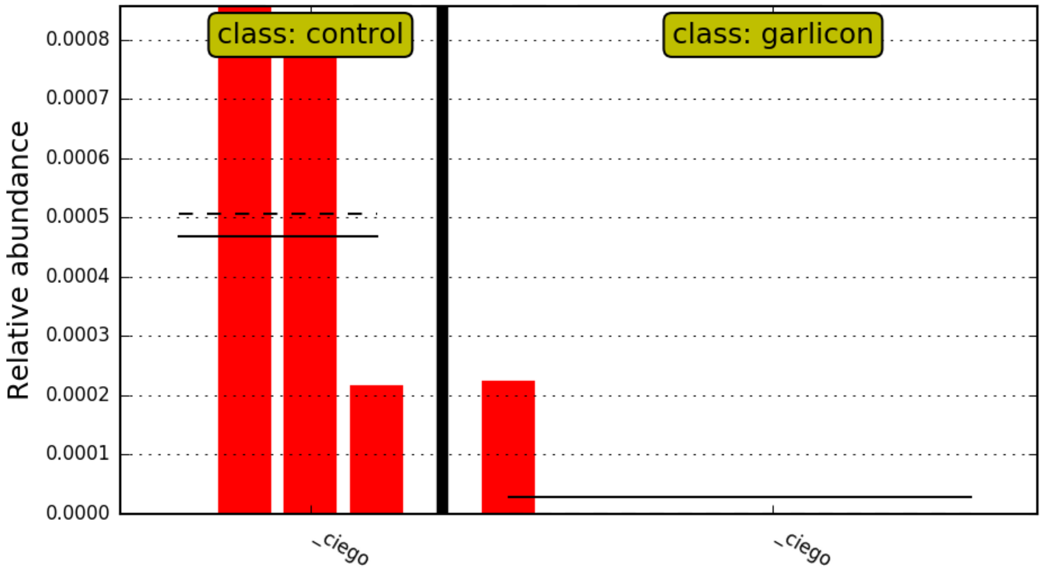

k__Bacteria_p__Proteobacteria_c__Gammaproteobacteria_o__Pseudomonadales_f__Moraxellaceae_g__Acinetobacter

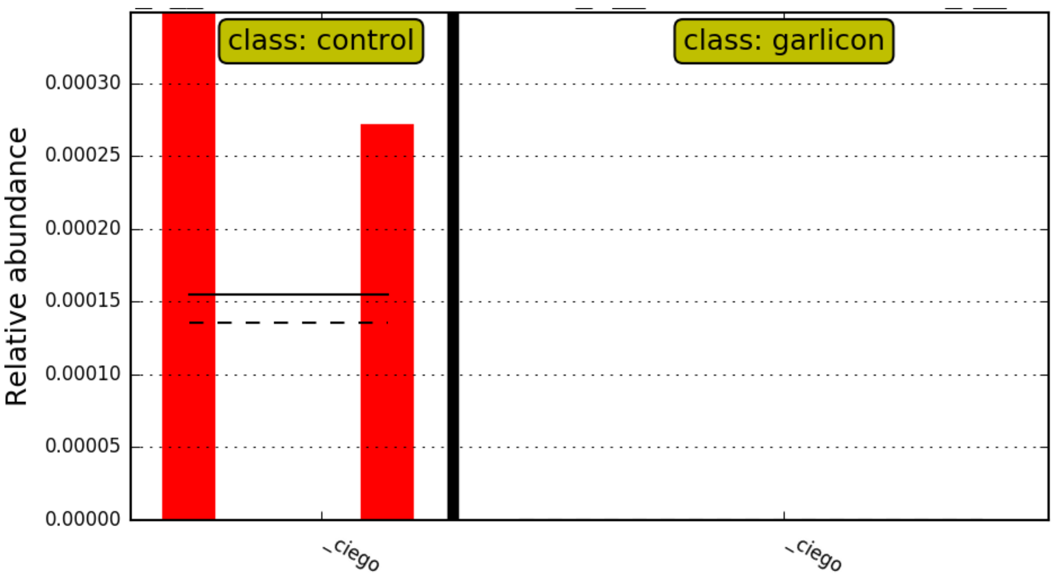

class: control
class: garlicon
Relative abundance
0.00030
0.00025
0.00020
0.00015
0.00010
0.00005
0.00000
_ciego
_ciego
